# Ocular and systemic bio-distribution of rhodamine-conjugated liposomes loaded with VIP injected into the vitreous of Lewis rats

**Published:** 2007-12-07

**Authors:** S. Camelo, L. Lajavardi, A. Bochot, B. Goldenberg, M.C. Naud, E. Fattal, F. Behar-Cohen, Y. de Kozak

**Affiliations:** 1 Center de Recherche des Cordeliers, Université Pierre et Marie Curie - Paris, Paris, France; 2 Univ Paris-Sud, CNRS, Physico-chimie - Pharmacotechnie - Biopharmacie, Faculté de Pharmacie, Châtenay-Malabry, France

## Abstract

**Purpose:**

Local delivery of therapeutic molecules encapsulated within liposomes is a promising method to treat ocular inflammation. The purpose of the present study was to define the biodistribution of rhodamine-conjugated liposomes loaded with vasoactive intestinal peptide (VIP), an immunosuppressive neuropeptide, following their intravitreal (IVT) injection in normal rats.

**Methods:**

Healthy seven- to eight-week-old Lewis male rats were injected into the vitreous with empty rhodamine-conjugated liposomes (Rh-Lip) or with VIP-loaded Rh-Lip (VIP-Rh-Lip; 50 mM of lipids with an encapsulation efficiency of 3.0±0.4 mmol VIP/mol lipids). Twenty-four h after IVT injection, the eyes, the cervical, mesenteric, and inguinal lymph nodes (LN), and spleen were collected. The phenotype and distribution of cells internalizing Rh-Lip and VIP-Rh-Lip were studied. Determination of VIP expression in ocular tissues and lymphoid organs and interactions with T cells in cervical LN was performed on whole mounted tissues and frozen tissue sections by immunofluorescence and confocal microscopy.

**Results:**

In the eye, 24 h following IVT injection, fluorescent liposomes (Rh-Lip and VIP-Rh-Lip) were detected mainly in the posterior segment of the eye (vitreous, inner layer of the retina) and to a lesser extent at the level of the iris root and ciliary body. Liposomes were internalized by activated retinal Müller glial cells, ocular tissue resident macrophages, and rare infiltrating activated macrophages. In addition, fluorescent liposomes were found in the episclera and conjunctiva where free VIP expression was also detected. In lymphoid organs, Rh-Lip and VIP-Rh-Lip were distributed almost exclusively in the cervical lymph nodes (LN) with only a few Rh-Lip-positive cells detected in the spleen and mesenteric LN and none in the inguinal LN. In the cervical LN, Rh-Lip were internalized by resident ED3-positive macrophages adjacent to CD4 and CD8-positive T lymphocytes. Some of these T lymphocytes in close contact with macrophages containing VIP-Rh-Lip expressed VIP.

**Conclusions:**

Liposomes are specifically internalized by retinal Müller glial cells and resident macrophages in the eye. A limited passage of fluorescent liposomes from the vitreous to the spleen via the conventional outflow pathway and the venous circulation was detected. The majority of fluorescent liposomes deposited in the conjunctiva following IVT injection reached the subcapsular sinus of the cervical LN via conjuntival lymphatics. In the cervical LN, Rh-Lip were internalized by resident subcapsular sinus macrophages adjacent to T lymphocytes. Detection of VIP in both macrophages and T cells in cervical LN suggests that IVT injection of VIP-Rh-Lip may increase ocular immune privilege by modulating the loco-regional immune environment. In conclusion, our observations suggest that IVT injection of VIP-loaded liposomes is a promising therapeutic strategy to dampen ocular inflammation by modulating macrophage and T cell activation mainly in the loco-regional immune system.

## Introduction

In the past few years, we have examined precisely the intraocular and systemic biodistribution of fluorescent antigens (Ag) injected into the anterior chamber (AC) of the rat eye. We and others have shown that fluorescent Ag is internalized predominantly by macrophages [1] located in the tissues lining the eye (such as the cornea, iris, ciliary body) and also by macrophages located in the conjunctiva [1,2]. We reported that soluble Ag reach the marginal zone of the spleen where they are internalized by resident ED3-positive macrophages via passage through the trabecular meshwork and Schlemm's canal [3]. In addition, we demonstrated that fluorescent Ag located in the conjunctiva is drained by conjunctival lymphatic vessels to cervical LN where it is captured by ED3-positive subcapsular sinus macrophages [3-5]. These macrophages are in contact with B and T lymphocytes and are thus potentially able to modulate the immune reactivity of these cells [3].

In humans, intravitreal (IVT) injection is routinely used for the intraocular delivery of therapeutic molecules [6,7]. However, to avoid repeated IVT injections, novel delivery systems are tested to improve safety and effectiveness [8-11]. Intraocular and systemic biodistribution of molecules injected intravitreally and the egress pathways of molecules originating from the vitreous could have important implications for the design of therapeutic strategies of ocular inflammation based on IVT injection.

Vasoactive intestinal peptide (VIP) [12] is a 28 amino acid immunomodulatory neuropeptide constitutively expressed in the eye and involved in the induction and maintenance of ocular immune privilege [13]. VIP modulates the activity of macrophages [14], dendritic cells [15,16], and T lymphocytes [17,18] in vitro and in vivo. Injection of VIP in vivo also induces the differentiation of regulatory dendritic cells and regulatory T cells [19,20]. Recently, we reported that IVT injection of VIP encapsulated in sterically-stabilized liposomes (VIP-Lip) could be used in immunosuppressive therapies of ocular inflammations [21].

The aim of the present study was to determine: (1) the intraocular and systemic biodistribution of empty rhodamine-conjugated liposomes (Rh-Lip) or VIP loaded Rh-Lip (VIP-Rh-Lip) 24 h following their IVT injection into healthy rats and (2) the nature of cells that internalize Rh-Lip and VIP-Rh-Lip in the eye and secondary lymphoid organs. Our results suggest that IVT injection of VIP-loaded liposomes could modulate the activation of macrophages and glial cells in the eye as well as macrophages and T lymphocytes in the cervical LN draining the ocular adnexae.

## Methods

### Animals

Eight-week-old male Lewis rats weighing around 280 g (R. Janvier, Le Genest Saint Isle, France) were used. Animals were maintained in a 12 h light, 12 h dark cycle. Food and water were supplied *ad libitum*. Three experiments using a total of 14 healthy rats were performed to study the biodistribution of rhodamine-conjugated liposomes (Rh-Lip) and rhodamine-conjugated VIP-loaded liposomes (VIP-Rh-Lip). Animals were handled and cared for in compliance with the ARVO Statement for the Use of Animals in Ophthalmic and Visual Research.

### Liposome preparation

rhodamine-conjugated pegylated (PEG) liposomes were composed of phosphatidylcholine (PC), phosphatidylglycerol (PG), cholesterol (Chol) and 1,2-distearoyl-*sn*-glycero-3-phosphoethanol-amine-*N*-[methoxy-poly -(ethyleneglycol)-2000] (PEG-DSPE) and phosphatidylethanolamine-*N*-(lissamine rhodamine B sulfonyl; PE-Rh). The final molar ratio was PC:PG:Chol:PEG-DSPE:PE-Rh (47:10:35:5:3, respectively). Liposomes were prepared by the thin-film hydration method as previously described [21,22].

### Physico-chemical characterization of liposomes

Liposome mean size was determined before and after encapsulation by quasi-elastic light scattering with a Nanosizer N4 Plus (Beckman Coulter, Margency, France) after having diluted the suspension 150 times in a saline solution. Measurement conditions were 20 °C, 1 mPa.s, refractory index of 1.33, 1 min data acquisition, and detection angle of 90°. Measurements made in triplicate were expressed as mean diameter±standard deviation. Liposomes presented a size between 300 and 600 nm for VIP-Lip and between 250 and 400 nm for unloaded Lip.

Zeta potential was measured using a Malvern Zetasizer Nano ZS (Malvern Instruments, Worcestershire, UK) at 25 °C following a 1/100 (v/v) dilution in a saline solution (150 mM). Both VIP-Lip and unloaded Lip presented slightly negative Zeta potential (−1.7 mV and −0.9 mV, respectively). Because of the high salt concentration, the distribution plot is not measurable.

The concentration of 28-aa VIP in liposomes was determined by enzyme linked immuno sorbent assay (ELISA); (VIP enzyme immunoassay (EIA) kit extraction free, Peninsula Laboratories Inc., San Carlos, CA). The principle of the assay is based on the competition between biotinylated VIP and non-biotinylated peptide (either standard or unknown) to bind on anti-VIP antibody that recognizes 28-aa VIP. Modifications were performed in the protocol to quantify VIP in liposome suspensions instead of in serum or plasma. Liposomes were solubilized by the addition of sodium dodecyl sulfate (SDS) 1% (1.06 mmol lipids/g SDS). Samples were then diluted in pH 7.7 buffer to reach VIP concentrations in a range of 0.1–10 ng/mL. The calibration curve was also prepared with pH 7.7 buffer instead of the standard diluent provided by the kit. Results were expressed as mean±standard deviation. VIP encapsulation yield was 37%±7%, corresponding to an encapsulation efficiency of 3.0±0.4 mmol VIP/mol lipids. Final VIP concentration within liposomes was 0.55 mg/mL.

### Intravitreal liposomes injections

To perform intravitreal injections, Lewis rats were anesthetized by intraperitoneal injection of 0.15 mL (5.47 g/100 ml saline) of Pentobarbital sodique® (Ceva santé animale, Libourne, France). Pupils were dilated by instillation of one drop of Tropicamide 5% (CibaVision Ophthalmics, Toulouse, France). One drop of Tetracaïne 1% (CibaVision Ophthalmics, Toulouse, France) was administered for local anesthesia. Intravitreal injections (IVT) of Rh-Lip (10 μl) were performed in both eyes using sterile syringes fitted with a 30-gauge needle (Microfine, Becton Dickinson AG, Meylan, France) as previously described [21].

### Tissue collection and processing for immuno-histochemistry

Immediately after sacrifice, the eyes, the right and left submandibular and superficial cervical LN (collectively named cervical LN), spleen, mesenteric LN, and inguinal LN were collected and processed for immunohistochemistry as previously described [21]. All tissues were post-fixed in 4% paraformaldehyde containing 5% sucrose for 2 h before being immersed for an additional 2 h in phosphate buffer containing 5% sucrose and finally overnight in phosphate buffer containing 15% sucrose. The next day samples were embedded and frozen in optimal cutting-temperature (OCT) compound (Tissue-Tek®, Sakura Finetek, Zoeterwoude, Netherland) and stored at −80 °C. Frozen sections (10 μm thick) of secondary lymphoid organs and antero-posterior sections of eyes at the optic nerve level were cut using a cryostat (Leica CM 3050S, Wetzlar, Germany) and mounted on gelatin-coated slides for immunohistochemical analysis.

### Immunohistochemistry

To characterize biodistribution of Rh-Lip containing cells (red), immunostaining was performed on the frozen eye and secondary lymphoid organ sections as previously described [1,21]. The slides were incubated with a range of purified mouse monoclonal antibody (mAb) all purchased from Serotec Ltd. (Oxford, UK) at dilution 1:50. T lymphocytes were detected with the mAb anti-T cell receptor (TCR; clone R73), the mAb anti-CD4 (clone W3–25) recognizing CD4-positive T cells and macrophages in rats [23] and the mAb anti-CD8 (clone OX8) against CD8-positive T cells. Macrophages were recognized by incubation with either the mAb anti-macrosialin CD68 specific of a cytoplasmic antigen in rat monocytes, macrophages and dendritic cells (clone ED1) or mAb anti-sialoadhesin, CD169 (clone ED3) [24]. Staining of dendritic cells was performed using the mAb OX-62 (clone OX-62). Secondary Alexa 488 (green)-conjugated goat-anti-mouse antibody (Invitrogen, Molecular Probes, Eugene, OR) was then applied at dilution 1:250. In some experiments, slides were counterstained with biotinylated-mouse anti-MHC class II mAb (clone OX6; Serotec Ltd.). The biotinylated mAb were revealed by incubation with Cy5 (infrared)-conjugated streptavidin (Interchim, Montluçon, France) at dilution 1:100. For VIP immuno-detection, sections were incubated with rabbit anti-VIP polyclonal antibody (Bachem AG (Merseyside, UK)) at dilution 1:100. Secondary Alexa 488-conjugated goat anti-rabbit mAb purchased from Interchim or Alexa 633-conjugated goat anti-rabbit mAb (Invitrogen, Molecular Probes) was then applied at dilution 1:250. Staining of Müller cells was performed using a rabbit polyclonal antibody against glial fibrilary acidic protein (1:100; GFAP) followed by incubation with Alexa 488-conjugated goat anti-rabbit mAb (Interchim) at dilution 1:250. In some experiments, the nucleus was detected by incubation with Toto3®-iodide, 1 mM in di-methyl sulfoxide (DMSO; Invitrogen, Molecular Probes). In every staining run, negative controls of immunostaining were performed by incubation of tissues sections with isotype control (mouse IgG1κ; purified protein, clone PMP; Serotec) followed by fluorochrome-conjugated mAb. All controls were negative. No rhodamine fluorescence was observed on tissue sections from animals that were not injected with Rh-Lip. Sections were mounted in phosphate buffered saline (PBS) containing glycerol (50:50, vol:vol).

### Confocal microscopy and image analysis

Confocal microscopy was performed using a Zeiss LSM 510 laser scanning confocal microscope equipped with an argon laser giving 488 nm (blue excitation, green emission), a helium/neon laser giving 543 nm (green excitation, red emission), and a helium neon laser giving 633 nm (green excitation, infrared emission), which allowed three fluorochromes to be observed. Sections were analyzed at 63X oil immersion objective (Zeiss plan-Apochromat NA 1.4) and at 40X objective (Zeiss plan neo fluar N.A 0.75); sequential images were merged and false colored using Zeiss LSM image browser software to produce a composite multicolor image. Final image processing was performed using Adobe Photoshop CS2 (Mountain View, Canada).

## Results

### Biodistribution of rhodamine-conjugated liposomes in ocular tissues and adnexae

The intraocular distribution of free Rh-Lip, VIP-Rh-Lip, and resident phagocytes containing liposomes 24 h post-IVT injection is summarized in Table 1. No difference in the biodistribution of cells containing Rh-Lip and VIP-Rh-Lip was observed. Cells containing fluorescent liposomes were mainly detected in the posterior segment of the eye along the retinal inner limiting membrane and in the vitreous. Liposomes were almost never detected in the retinal parenchyma per se and Rh-Lip were not observed at the level of the choroid or sclera. However, free rhodamine was detected within the internal layers of the retina, suggesting that a release of free VIP in the retinal parenchyma could occur as well. In the anterior segment of the eye, only a few cells containing Rh-Lip were seen in the ciliary body. The iris and cornea were devoid of free Rh-Lip and of cells containing Rh-Lip. At the level of ocular adnexae, many free Rh-Lip and cells containing Rh-Lip were located in the conjunctiva and the episclera.

**Table 1:** Distribution of Rh-Lip remains constant over time.

**Tissues containing RH-Lip (# Positive/# Tested)**					
	**Injected eye**	**CLN**	**Spleen**	**MLN**	**ILN**
**24 Hours**	3/3	3/3	3/3*	1/3*	0/3
**14 Days**	2/2	1/2	1/1*	0/2	0/2

Biodistribution of Rh-Lip and VIP-Rh-Lip bearing cells 24 h following IVT injection.

### Phenotypic characterization of intraocular and conjunctival phagocytes internalizing rhodamine-conjugated liposomes (Rh-Lip or VIP-Rh-Lip)

**Figure 1:**
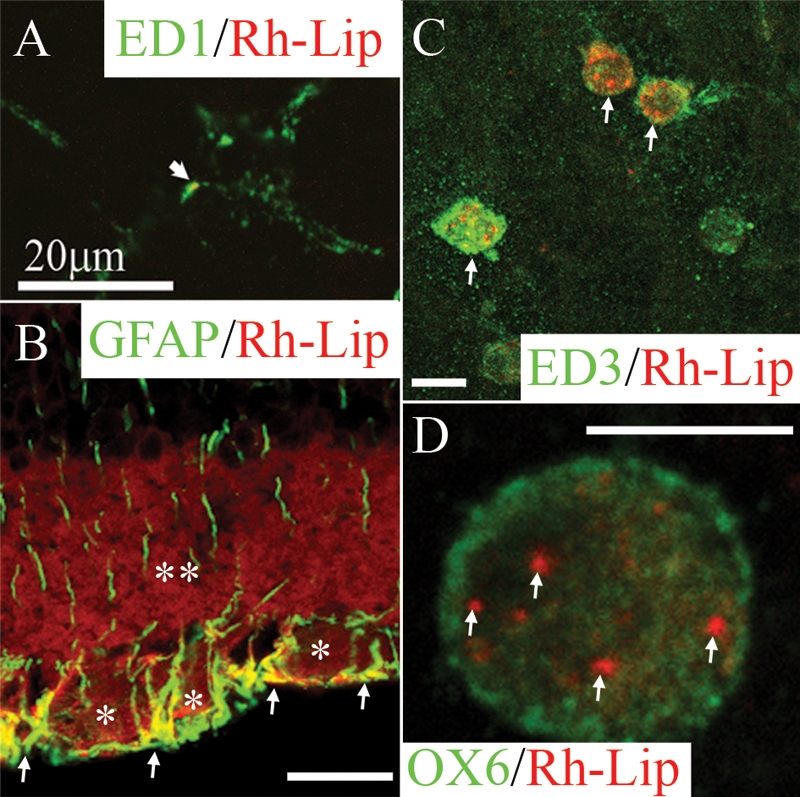
Biodistribution and phenotype of cells internalizing rhodamine-conjugated liposomes in the retina and vitreous of normal rats 24 h post IVT injection of Rh-Lip. **A**: On a whole-mounted retina, some microglial cells stained with ED1 (green) are detected phagocytosing limited number of rhodamine-conjugated-liposomes (Rh-Lip, red). **B**: rhodamine-conjugated-liposomes (Rh-Lip, red) are internalized by GFAP-positive (green)-retinal Müller glial cells as evidenced by colocalization (yellow) in the internal limiting membrane and the ganglion cell layer. Fluorescent Rh-Lip are seen as red fluorescent round dots on the images while free rhodamine is detected as diffuse red fluorescence within the ganglion cell layer (an asterisk) and inner plexiform layer (a double asterisk). **C**: On a whole-mounted retina, macrophages expressing ED3 (green) located at the level of the inner limiting membrane of the retina internalized Rh-Lip injected into the vitreous. **D**: Some of these macrophages express OX6 (green) suggesting they are activated and contain various amount of Rh-Lip. **D** shows a single OX6-positive macrophage containing a few Rh-Lip located into its cytoplasm. Photographs are representative of images obtained from frozen sections (**B**) and whole mounts (**A**, **C**, and **D**) performed on a total of 12 eyes. All bars represent 20 μm except in **D** (10 μm). Confocal optical section, in all images, is 3 μm.

At the level of the internal retina, rare liposomes were internalized by ED1-positive ramified microglial cells (Figure 1A). More often Rh-Lip were internalized by activated GFAP-positive Müller glial cells (Figure 1B). In the vitreous and along the inner limiting membrane of the retina, rare infiltrating cells had taken up liposomes. This observation indicated that liposomes have a modest proinflammatory effect. The cells internalizing liposomes in the vitreous presented a rounded morphology and were mostly ED1-positive (data not shown) and ED3-positive (Figure 1C). A few cells containing Rh-Lip also expressed MHC class II molecules (OX6; Figure 1D), suggesting that they correspond to activated macrophages and are capable of Ag presentation. The amount of Rh-Lip within the cytoplasm of these phagocytes was variable; some cells contained only a low number of Rh-Lip (Figure 1D) while others OX6-positive cells had their cytoplasm filled with fluorescent liposomes (data not shown).

**Figure 2:**
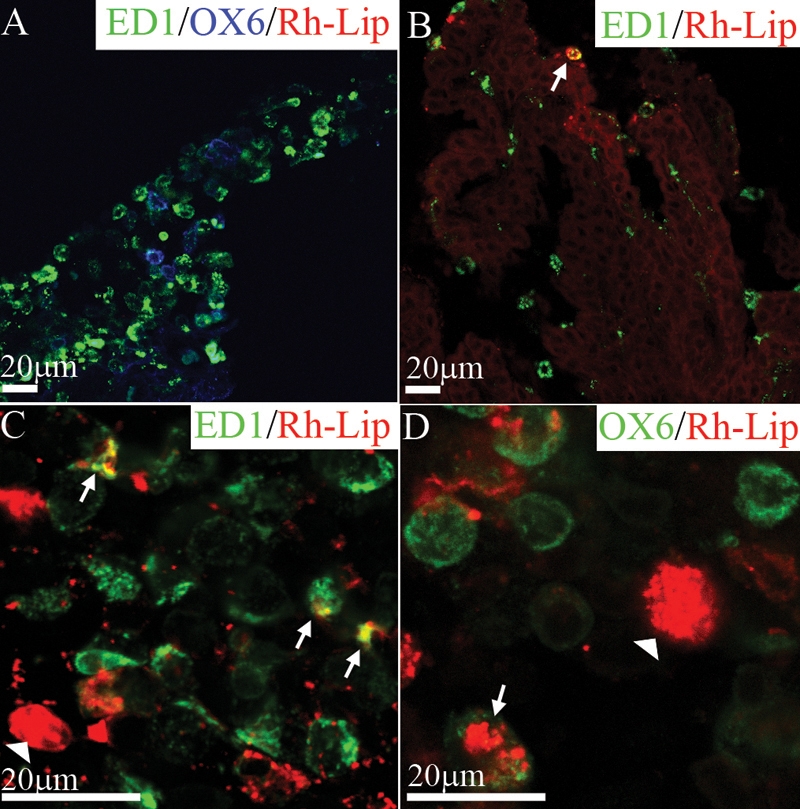
Biodistribution and phenotype of cells internalizing rhodamine-conjugated-liposomes in the iris and ciliary body of normal rats 24 h post IVT injection of Rh-Lip. **A**: On a frozen section of the iris, resident iridal ED1-positive (green) and OX6-positive cells (blue) are negative for fluorescent liposomes. **B**: Frozen section of the ciliary body showing a rare ED1-positive cell containing liposomes (arrow), ED1 green, rhodamine-conjugated-liposomes red, colocalization yellow. **C**: Ciliary body flat mount with ED1-positive cells containing fluorescent liposomes (arrows). The white arrowhead shows liposomes internalized by an ED1-negative cell. Other fluorescent liposomes appear not to be internalized by cells (red dots). **D**: Uptake of rhodamine-conjugated-liposomes by OX6-positive cells (green) appears limited to a few cells (arrow), the arrowhead shows liposomes internalized inside an OX6-negative cell. Photographs are representative of images obtained from frozen sections (**A**,**B**) and whole mounts (**C**,**D**) performed on a total of 12 eyes. All bars represent 20 μm. Confocal optical section, in all images, is 1.5 μm.

In the anterior segment, no uptake of Rh-Lip by ED1 or OX6-positive cells on the iris was detected (Figure 2A). By contrast, on the ciliary body, some cells contained Rh-Lip (Figure 2B). These cells expressed mostly ED1 (Figure 2C) but rarely MHC class II (OX6; arrow in Figure 2D). A limited number of infiltrating inflammatory cells were detected in the aqueous humor confirming the low inflammatory potential of Rh-Lip.

**Figure 3:**
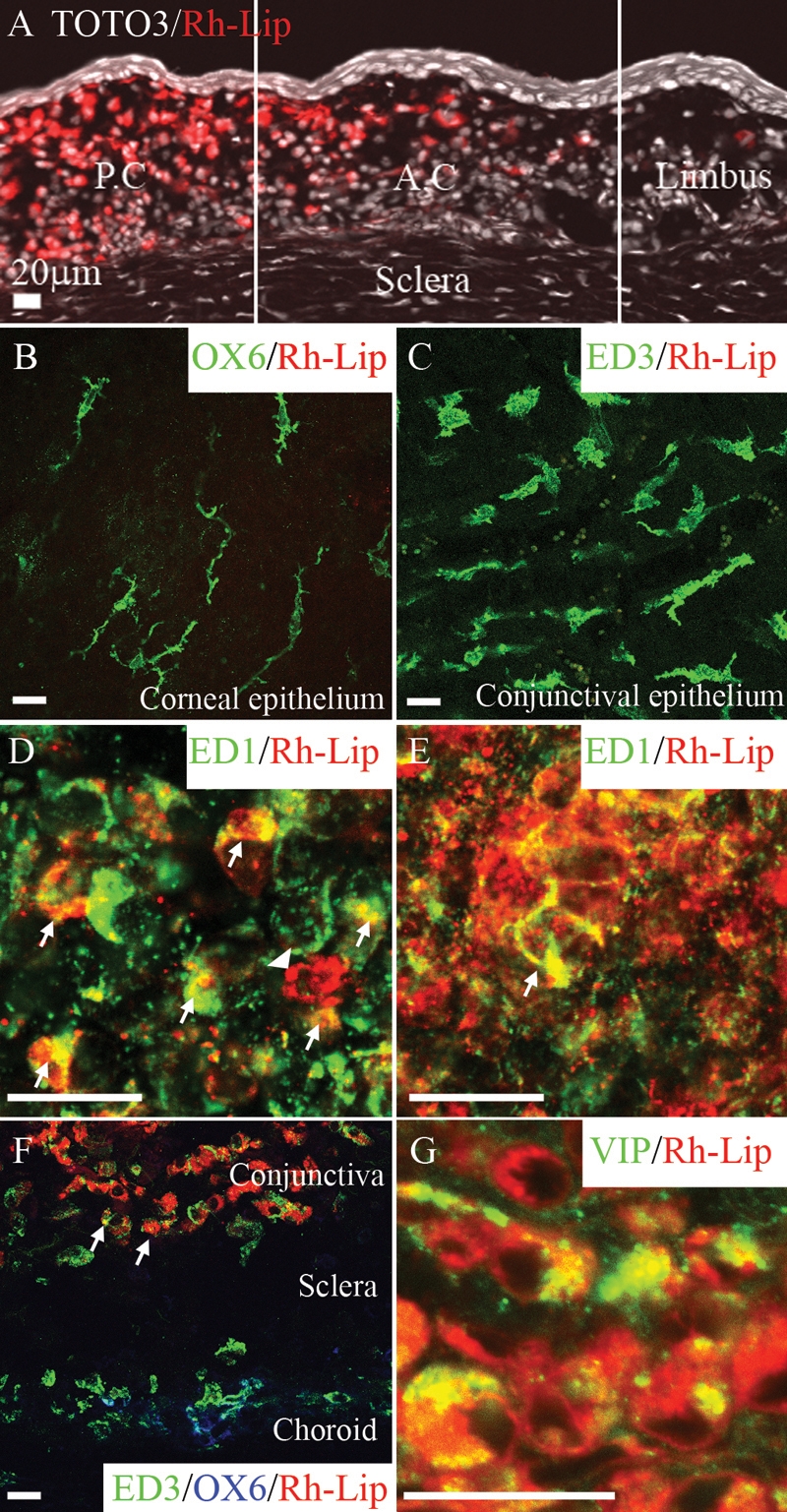
rhodamine-conjugated-liposomes and VIP biodistribution in conjunctiva 24 h following IVT injection of Rh-Lip and VIP-Rh-Lip. **A**: Following IVT injection, Rh-Lip (red) are internalized by cells in the conjunctival stroma. Fluorescent liposomes are not detected in conjunctival epithelium and sclera. Liposome concentration is maximal in the posterior conjunctiva (P.C) near the site of injection and decreases toward the anterior conjuntiva (A.C.) and the limbus. No liposomes were detected in the corneal stroma (data not shown). Nuclei stained with Toto3®-iodide are depicted in white. **B**: OX6-positive dendritic cell in the epithelium and **C**: ED3-positive macrophages in the conjunctival epithelium do not internalize Rh-Lip. **D**: ED1-positive cells in the anterior conjunctival stroma and **E**: in the posterior conjunctival stroma contain large amount of Rh-Lip. **F**: Fluorescent Rh-Lip (red) in the conjunctival stroma are taken up by ED3-positive (green), OX6 (blue)-negative macrophages (arrows). Choroidal ED3 and OX6-positive cells do not contain liposomes. **G**: VIP expression in the conjunctiva of a rat that received an IVT injection of VIP-Rh-Lip. VIP, detected with rabbit anti-VIP antibody (green) is localized in cells containing Rh-Lip (red) in the conjunctiva. Note the extra cellular green dots representing free VIP not internalized by cells. VIP appears green, Rh-Lip appears red and colocalization is indicated in yellow. All bars represent 20 μm, confocal microscopy optical section is 1.5 μm in all images. Photographs are representative of images obtained from frozen sections (**A**,**F**,**G**) and whole mounts (**B**-**E**) performed on a total of 12 eyes except **G** that is a representative image of ocular frozen sections from four eyes.

There was a quantitative gradient of Rh-Lip located in the ocular adnexae. The highest concentration of fluorescent liposomes was detected in the posterior conjunctiva (P.C) near the site of the injection and decreased gradually toward the posterior episclera and anterior conjunctiva (A.C, Figure 3A). At the level of the cornea, no Rh-lip could be detected (not shown). No fluorescent liposomes were detected in the sclera and corneal and conjunctival epitheliums. Langerhans-like MHC class II-positive cells in the corneal epithelium (Figure 3B) and ED3-positive macrophages in the conjunctival epithelium did not contain fluorescent liposomes (Figure 3C). rhodamine-conjugated liposomes were located exclusively in the conjunctival stroma (Figure 3A) in ED1-positive macrophages in the posterior (Figure 3D) and anterior (Figure 3E) conjunctival stroma. In addition, ED3-positive macrophages containing Rh-Lip were MHC-Class II (OX6)-negative (Figure 3F). Fluorescent liposomes internalized by macrophages in the conjunctiva still contained VIP 24 h following IVT injection of VIP-Rh-Lip, (Figure 3G), but VIP was not detected in conjunctival macrophages after IVT injection of Rh-Lip alone (data not shown). In the choroid, MHC class II-positive dendritic cells did not contain any fluorescent liposomes (Figure 3F). The nature and distribution of cells internalizing rhodamine-conjugated liposomes in the eye was equivalent when checked at 24 h, seven days (data not shown), and 14 days following IVT injection (Table 2).

**Table 2:** Ocular distribution of Rh-Lip and VIP-Rh-Lip are equivalent

**Tissue**	**Rh-Lip n=3**	**VIP-Rh-Lip n=3**
Aqueous humor	-	-
Vitreous	++	++
Inner limiting membrane	++	++
Retinal parenchyma	-	-
Iris	-	-
Ciliary body and muscles	+	+
Conjunctiva	++	++
Episclera	++	++

Ocular and systemic distribution of Rh-Lip-bearing cells 24 h and 14 days following their IVT injections

### Distribution of Rh-Lip in the secondary lymphoid organs following intravitreal injection of Rh-Lip

Following IVT injection of Rh-Lip, fluorescent liposomes were observed in cervical LN as early as 2 h after IVT injection (data not shown). Twenty-four h and up to 14 days following injection, Rh-Lip was almost exclusively detected in the subcapsular sinus of the cervical LN (Table 2). In several places, red fluorescence was detected in the B cell area of the cervical LN but was not associated with a specific cell type (data not shown). We hypothesize that this red fluorescence corresponds to a leakage of rhodamine from adjacent Rh-Lip. A rare cell containing Rh-Lip was observed in the subcapsular sinus of the mesenteric LN of one animal out of three (Table 2), and only a few cells bearing a limited quantity of Rh-Lip were detected in the marginal zone of the spleen. No difference was noted in the biodistribution of unloaded liposomes (Rh-Lip) and liposomes containing VIP (VIP-Rh-Lip; Table 2).

### Localization and phenotypic characterization of Rh-Lip-bearing cells in the secondary lymphoid organs

**Figure 4:**
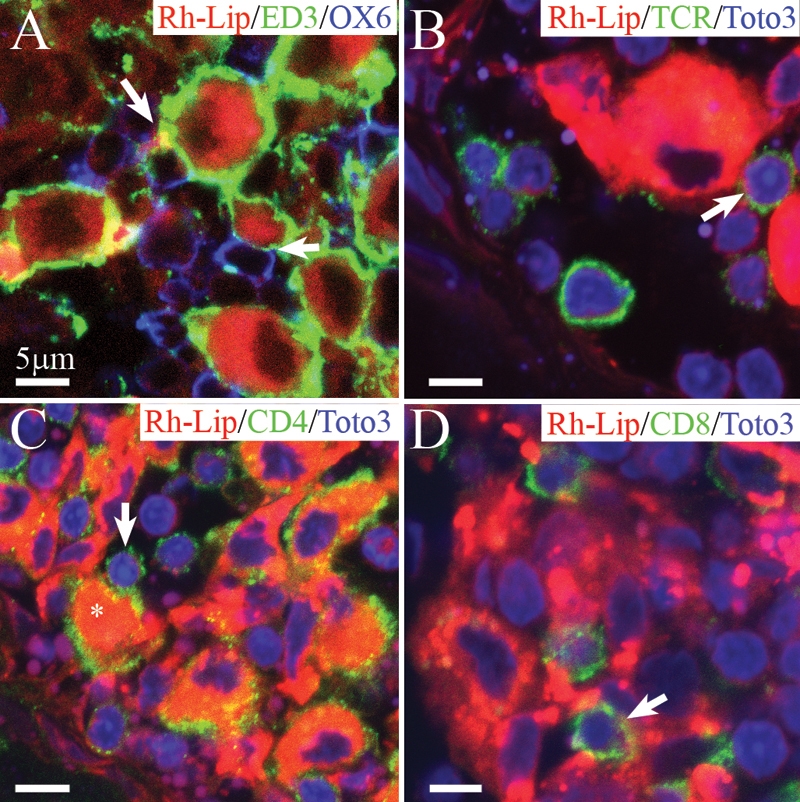
Biodistribution of rhodamine-conjugated-liposomes in cervical LN in healthy rats 24 h following IVT injection of VIP-Rh-Lip. **A**: Following IVT injection, VIP-Rh-Lip (red) are located within subcapsular sinus macrophages stained with ED3 (green). OX6-positive cells in blue are located in close proximity (arrow) with ED3-positive macrophages containing VIP-Rh-Lip. **B**: T lymphocytes recognized by their TCR expression (green) are localized in contact (arrow) with subcapsular macrophages containing VIP-Rh-Lip (red). C: Subcapsular sinus macrophages (asterisk) containing VIP-Rh-Lip (red) express CD4 in green and are in contact with CD4-positive lymphocytes (arrow). **D**: CD8-positive T lymphocytes (green) are also adjacent with VIP-Rh-Lip (red)-bearing macrophages. In **B**, **C**, and **D** nuclei are stained in blue with Toto3®-iodide. All bars represent 5 μm. Confocal microscopy optical sections are 1.5 μm in all images. Representative images of four experiments performed on cervical LN from four rats.

To characterize the nature of the cells that have internalized Rh-Lip in the secondary lymphoid organs 24 h following their IVT injection, cervical LN and spleen sections were subjected to immunofluorescent-staining with a range of mAbs specific for phenotypic markers of macrophages and dendritic cells. The rare Rh-bearing cells were located in the mesenteric LN and in the marginal zone of the spleen expressed ED3 (data not shown), confirming their identity as resident macrophages. In the cervical LN, Rh-Lip-bearing cells were situated mostly just underneath the subcapsular sinus. Some of these cells were also found more deeply within the T cell area and around B cell follicles. These cells expressed the macrophage markers, ED1 (data not shown) and ED3 (Figure 4A). These cells failed to express the dendritic cell marker OX62 (data not shown), strongly indicating they are subcapsular sinus macrophages and not dendritic cells [24]. In addition, free rhodamine was observed in the cervical LN at the level of some B cell follicles, which are generally surrounded by ED3-positive macrophages (not shown). This suggests that liposome degradation products generated by subcapsular sinus macrophages may become available to B lymphocytes.

In the cervical LN, we observed numerous small, round OX6-positive cells in contact with ED3-positive macrophages containing Rh-Lip (Figure 4A). Subcapsular sinus macrophages surround B cells follicles thus these OX6-positive cells in contact with Rh-Lip-bearing macrophages are most probably B cells. In addition, we detected TCR-positive T lymphocytes in close proximity with cells that had internalized Rh-Lip (Figure 4B). Many cells of small size adjacent to Rh-Lip-containing macrophages expressed CD4 (Figure 4C). Of note, CD4 is also expressed by subcapsular sinus macrophages (Figure 4C, asterisk) as previously reported for all macrophages in the rat [23]. Cells expressing CD8 were also observed to be adjacent to Rh-Lip-bearing macrophages (Figure 2D). No difference was noted regarding the nature of the cells internalizing liposomes and the cellular interactions with B cells and T cells in the LN in rats receiving Rh-Lip and VIP-Rh-Lip, respectively.

### VIP biodistribution in cervical lymph nodes following an intravitreal injection of VIP-Rh-Lip

**Figure 5:**
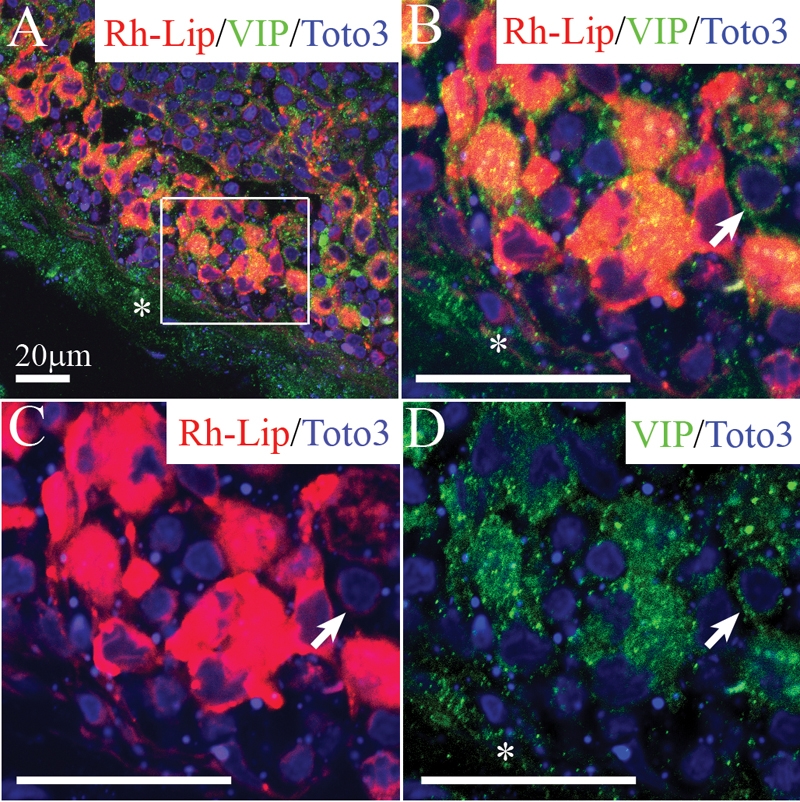
VIP biodistribution in cervical LN 24 h following IVT injection of VIP-Rh-Lip. **A**: Free VIP (green), detected with rabbit anti-VIP antibody is localized in the sinus (asterisks) and within subcapsular sinus macrophages containing rhodamine-conjugated-liposomes (red; **B** to **D**) Enlargement of the inset in **A** is shown in **B** to **D**. Free soluble VIP (green) is detected as green dots in the LN capsule (asterisks) and the LN parenchyma. VIP (appearing as yellow granules) is also detected within resident macrophages containing Rh-Lip (red cells in **B** and **C**). VIP is detected in small round cells that contain only minute amounts of Rh-Lip (arrows in **B**, **C**, and **D**). In **A** to **D** VIP appears green, Rh-Lip appears red and colocalization is indicated in yellow. Nuclei are stained in blue with Toto3®-iodide.All bars represent 20 μm, confocal microscopy optical section is 1.5 μm in all images. Representative images of two experiments performed on cervical LN from two rats.

**Figure 6:**
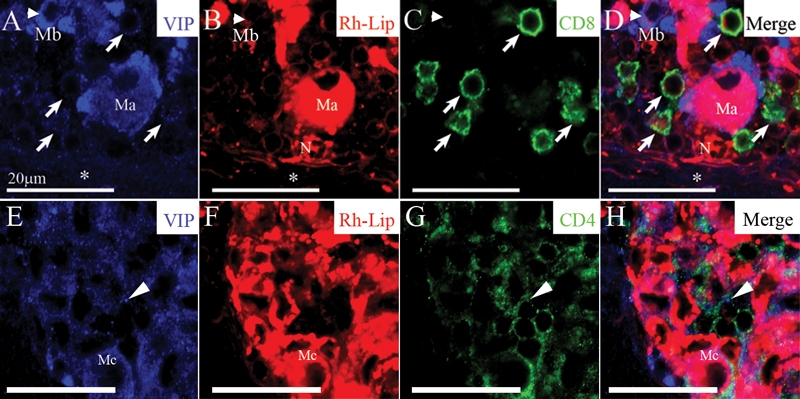
VIP detection in CD4 and CD8-positive T-cells in the cervical LN of rats receiving an IVT injection of VIP-loaded-rhodamine-conjugated-liposomes. **A**-**D**: Immuno-detection of VIP as blue dots in the LN capsule (asterisk), the LN parenchyma, within VIP-Rh-Lip-bearing macrophage (Ma) but also within macrophages that do not contain VIP-Rh-Lip (Mb, arrowhead): compare the localization of VIP in **A**, with the localization of VIP-Rh-Lip in red (**B**). VIP is present at the membrane of CD8-positive T lymphocytes in green (arrows): compare image **A** and **C**. Note the presence of a neutrophil (N) in **B** and **D** containing Rh-Lip in contact with macrophage Ma. VIP is present in small cells expressing CD4 in green (arrows; **G**,**H**) in the vicinity of macrophages containing Rh-Lip (Mc; **E**,**F**). In **A** to **H**, VIP is in blue, Rh-Lip in red, T cell markers in green and colocalization is in purple. All bars represent 20 μm, confocal microscopy optical section is 1.5 μm in all images. Representative images of two experiments performed on cervical LN from two rats.

**Figure 7:**
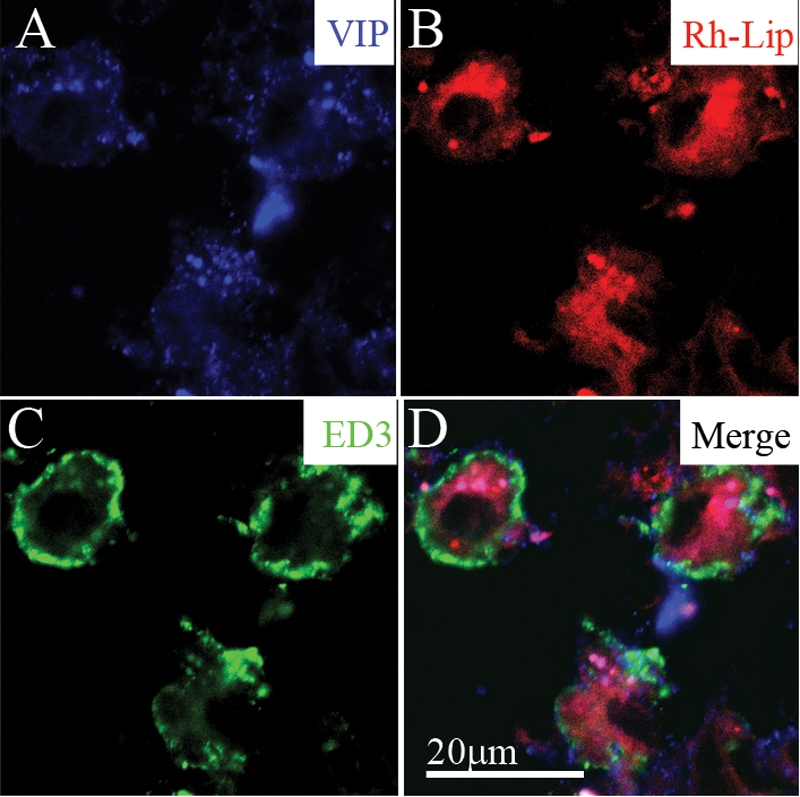
VIP-loaded rhodamine-conjugated-liposomes (VIP-Rh-lip) internalization and VIP expression by ED3-positive macrophages in cervical LN 24 h following IVT injection of VIP-Rh-Lip. **A**: Free VIP, detected with rabbit anti-VIP antibody (blue) localized within cells containing Rh-Lip (red; **B**) and expressing ED3 green (**C**). **D**: Merge image showing membranous expression of ED3 by cells containing Rh-lip and blue granules within liposomes. The bar in **D** represents 20 μm in all images. Confocal microscopy optical section is 2 μm in all images. Representative images of two experiments performed on cervical LN from two rats.

VIP immunostaining was performed on frozen sections of cervical LN of normal animals that received a single IVT injection of VIP-Rh-Lip. Soluble VIP (green) was detected in the sinus of the LN (asterisks in Figure 5A,B,D). In addition, VIP was detected within large cells containing Rh-Lip corresponding to subcapsular sinus macrophages (Figure 5A-D). VIP was also detected in small, round cells (characterized in Figure 6) that contained only a minute amount of Rh-Lip (arrow in Figure 5B-D) close to VIP-Rh-Lip-bearing macrophages in the subcapsular sinus. In animals that received an IVT injection of Rh-Lip that did not contain VIP, there was no VIP detectable in the subcapsular sinus of the cervical LN (data not shown). These macrophages expressed ED3 confirming their identity as subcapsular sinus macrophages (Figure 7A-D). Our results suggest strongly that VIP travels encapsulated within liposomes from the conjunctiva (not shown) to the cervical LN (Figure 5A) where it is phagocytosed by resident macrophages.

To determine the nature of the small cells adjacent to macrophages in the cervical LN in which VIP was detected, triple immunostaining for VIP (blue), Rh-Lip (red), and T cell markers (green) were performed. VIP was detected in the capsular sinus (asterisk in Figure 6A,B,D) and the LN parenchyma (Figure 6A,B,D). VIP was also detected within VIP-Rh-Lip-bearing macrophages (Ma and Mb in Figure 6A,B,D) and in CD8-positive T cells (arrows in Figure 6A,C,D). In addition, VIP was present in macrophages (Mc, Figure 5E) containing Rh-Lip (Figure 6F) and in small cells expressing CD4 (arrow in Figure 6G,H). This result strongly suggests that the small cells immuno-positive for VIP are CD4 and CD8-positive T lymphocytes. The T lymphocyte nature of these cells was confirmed by their expression of TCR (data not shown).

## Discussion

Liposome encapsulation of pharmaceutical molecules is of great interest for efficient drug delivery to intraocular tissues [8-11]. However, distribution studies of liposomes are needed to optimize ocular disease therapies. In the present study, we show that following IVT injection in healthy Lewis rats, free rhodamine-conjugated liposomes (Rh-Lip) and cells bearing liposomes were located mainly in tissues of the posterior segment of the eye, namely the vitreous and inner layers of the retina. The distribution of Rh-lip in the eye was equivalent at 24 h, seven days, and 14 days (Table 2 and data not shown), but the amount of intraocular liposomes decreased with time.

We noted the presence of free rhodamine within the internal layers of the retina suggesting that a release of free VIP could occur at this level. Indeed, we have shown previously [21] that VIP released from liposomes at the level of the inner retina enters the retina passively, potentially modulating the immunological properties of both retinal Müller glial cells and microglia.

In the present study, an uptake of Rh-Lip by activated retinal Müller glial cells was observed. Müller glial cells are giant cells spanning the full thickness of the retina from the internal to the external limiting membranes, their end feet lie along the internal limiting membrane in close contact with the vitreous and the retinal blood vessels. These cells have been shown to be involved in modulation of the ocular immune responses during uveitis [25,26]. Moreover, Müller glial cells transduced ex vivo with adenovirus expressing viral IL-10 (vIL-10) and injected IVT to recipient animals, induced the expression of vIL-10 in ocular tissues and media, and reduced the pathological manifestations of experimental autoimmune uveoretinitis (EAU) [27]. We hypothesize that liposome uptake by retinal Müller glial cells could contribute to extended intraocular drug delivery and participate in the modulation of ocular inflammation. In contrast, liposomes were poorly internalized by microglia, by cells located in the choroid, or by retinal pigmented epithelial (RPE) cells. This observation is consistent with our previous findings showing that following IVT injection of liposomes loaded with oligonucleotides in rabbits, more than 70% of the oligonucleotides were detected at 24 h in the vitreous while less than 5% was detected in the retina choroid, lens, and sclera [28]. The limited retinal penetration by liposomes is either due to their size or pharmaceutical properties. The size of liposomes used in this study varies between 250 and 400 nm for Rh-Lip and 300 and 600 nm for VIP-Rh-Lip [21]. Smaller liposomes (less than 100 nm) can easily be produced, but VIP encapsulation in these small liposomes by the lyophilization-rehydration method leads to the increase of liposome size. Other encapsulation methods were unfeasible because it destroyed VIP [22]. To optimize therapeutic strategies based on IVT injection of liposomes, we propose to inject a mix of different size liposomes containing various therapeutic molecules. This could result in the specific targeting of macrophages in the vitreous and Müller cells at the level of the inner limiting membrane of the retina by VIP-loaded large liposomes (300–600 nm). At the same time, other molecules encapsulated in small liposomes (less than 100 nm) could reach the outer segments of the retina and the RPE and thus protect photoreceptors from degeneration. Pharmacological and therapeutic studies need to be performed to determine the efficacy of this method.

We have previously shown that intravitreal injection of liposomes loaded with VIP successfully reduced ocular inflammation induced by subcutaneous injection of LPS (endotoxin-induced uveitis, EIU), a non-specific ocular inflammation of short duration [21]. In the present paper, we have checked with precision the ocular and immune system biodistribution of rhodamine-conjugated liposomes in healthy animals. No difference in distribution in the lymphoid organs was found between healthy animals and EIU rats [21]. Moreover, biodistribution of rhodamine-conjugated liposomes in the posterior segment of the eye was identical in healthy rats as shown in the present work and in EIU rats [21]. However, in animals with EIU, liposomes were also internalized in the anterior segment by infiltrating neutrophils and macrophages. This phenomenon is probably partly responsible for the therapeutic effect of the IVT injection of VIP-Lip during EIU [21]. In contrast, only few liposomes were detected in the anterior segment of healthy rat eyes where there are no inflammatory cells.

The intraocular biodistribution of rhodamine-conjugated liposomes following their IVT injection reported here differs widely from the biodistribution of fluorescent Ag injected into the AC of the eye reported previously [1,3,5]. Indeed, following an intracameral injection, fluorescent Ag were detected in cells located preeminently in tissues lining the AC of the eye, namely the cornea, iris, and ciliary body [1,3,5]. Ag then reached the venous circulation via the trabecular meshworks and Schlemm's canal, and a large quantity of fluorescent Ag was captured by macrophages in the marginal zone of the spleen and mesenteric LN [1,3-5]. However, in the present study, very few liposomes injected into the vitreous were detected, either free or taken up by cells, in the anterior segment of the eye, and only rare macrophages containing liposomes were observed in the spleen. We propose that only limited amounts of liposomes were drained out of the vitreous via the AC and Schlemm's canal (conventional aqueous outflow pathway), and thus very few liposomes reached the marginal zone of the spleen of IVT injected rats. We have previously shown that polystyrene and cyanoacrylate nanoparticles [29] injected into the vitreous were able to enter the anterior segment of the eye, to accumulate in Schlemm's canal, and to leave the eye via the venous circulation. This suggests that size and physico-chemical properties of molecules and particles injected into the vitreous influence their egress pathway from the eye.

In the present study, we found large amounts of IVT-injected Rh-Lip internalized by macrophages in the conjunctiva and then in subcapsular macrophages in the cervical LN even 14 days following the IVT injection ([21] and Table 2). We have previously demonstrated that Ag placed into the AC of the eye can access the conjunctiva via the nonconventional aqueous outflow pathway and from there reach the cervical LN via conjunctival lymphatic vessels [30,31]. In the present study, Rh-Lip drainage to the cervical LN more probably results from Rh-Lip deposition in the conjunctival tissue occurring during the IVT injection itself.

In summary, the present fluorescent liposome biodistribution study shows that (1) most liposomes stay in the vitreous and show limited penetration in the retina where they are mostly internalized by retinal Müller glial cells but not by microglia; (2) only few Rh-Lip placed into the vitreous body leave the eye via the conventional outflow pathway through Schlemm's canal, and consequently, few reach the venous circulation and are internalized by marginal zone macrophages in the spleen; and (3) the fluorescent liposomes deposited in the conjunctiva during IVT injection access the cervical LN via lymphatic vessels in the conjunctiva.

As previously reported following intracameral injection of fluorescent Ag, VIP-Rh-Lip were internalized in the cervical LN by subcapsular sinus macrophages expressing ED3 [4,5,32]. The VIP-loaded macrophages are in contact with OX6 (probably B cells) and TCR-, CD4- or CD8-positive T cells in the cervical LN. No liposome uptake by OX62-positive dendritic cells in the cervical LN was observed (data not shown). Internalization of VIP-Rh-Lip by macrophages in the cervical LN in vivo could alter and modify their function from inflammatory macrophages to regulatory macrophages. Moreover, macrophages internalizing VIP-Rh-Lip could participate in the slow release of VIP in the LN, possibly changing the LN immune environment over a long period of time. A large quantity of VIP was detected as free molecules in the subcapsular sinus of the LN. VIP observed within the LN parenchyma was also probably associated as free peptide with the reticular network of high endothelial venules in the LN as previously described following subcutaneous injection of small molecules and chemokines [31,33]. Free VIP associated with the reticular network of high endothelial venules in the LN can modulate immune function of macrophages, dendritic cells, and B and T lymphocytes [18]. In the cervical LN of VIP-Rh-Lip injected rats, VIP was detected in T lymphocytes. VIP diffusing from VIP-Rh-Lip contained in ED3-positive macrophages could induce VIP production in adjacent T cells. Indeed, T cells express VPAC1 and VPAC2, two of the three VIP receptors [34]. Thus, VIP detected in T lymphocytes could either represent VIP attached to its membranous receptor or VIP produced by T cells in response to VIP stimulation as previously reported [35]. VIP immunoreactivity in macrophages and T cells in the cervical LN suggests that a single IVT injection of liposomes loaded with VIP could modulate the loco-regional ocular immune response and thus reinforce the ocular immune privilege [13].

In conclusion, an IVT injection of VIP encapsulated within liposomes provides long-term expression of VIP inside the eye and in the cervical LN draining the eye. The resulting potential modulation of the loco-regional immune microenvironment suggests that a single IVT injection of VIP-Lip is a rational therapeutic modality for posterior uveitis and other immune-mediated ocular diseases in humans.
